# Enhancement of ascomycin production via a combination of atmospheric and room temperature plasma mutagenesis in *Streptomyces hygroscopicus* and medium optimization

**DOI:** 10.1186/s13568-019-0749-x

**Published:** 2019-02-18

**Authors:** Zhituo Yu, Xiaofang Shen, Yuanjie Wu, Songbai Yang, Dianwen Ju, Shaoxin Chen

**Affiliations:** 1 Department of Microbiological and Biochemical Pharmacy, The Key Laboratory of Smart Drug Delivery, Ministry of Education, School of Pharmacy, Fudan University, Shanghai, 201203 China; 2 Shanghai Institute of Pharmaceutical Industry, China State Institute of Pharmaceutical Industry, Shanghai, 201203 China

**Keywords:** *Streptomyces hygroscopicus*, Ascomycin, Atmospheric and room temperature plasma, Response surface methodology, Medium optimization

## Abstract

Ascomycin, a key intermediate for chemical synthesis of immunosuppressive drug pimecrolimus, is produced by *Streptomyces hygroscopicus* var. *ascomyceticus*. In order to improve the strain production, the original *S. hygroscopicus* ATCC 14891 strain was treated here with atmospheric and room temperature plasma to obtain a stable high-producing *S. hygroscopicus* SFK-36 strain which produced 495.3 mg/L ascomycin, a 32.5% increase in ascomycin compared to the ATCC 14891. Then, fermentation medium was optimized using response surface methodology to further enhance ascomycin production. In the optimized medium containing 81.0 g/L soluble starch, 57.4 g/L peanut meal, and 15.8 g/L soybean oil, the ascomycin yield reached 1466.3 mg/L in flask culture. Furthermore, the fermentation process was carried out in a 5 L fermenter, and the ascomycin yield reached 1476.9 mg/L, which is the highest ascomycin yield reported so far. Therefore, traditional mutagenesis breeding combined with medium optimization is an effective approach for the enhancement of ascomycin production.

**Electronic supplementary material:**

The online version of this article (10.1186/s13568-019-0749-x) contains supplementary material, which is available to authorized users.

## Introduction

Ascomycin, a 23-membered macrocyclic polyketide (Additional file 1: Figure S1), was produced by *Streptomyces hygroscopicus* var. *ascomyceticus* ATCC 14891 strain (Bérdy 2005). Ascomycin is pharmacologically important, because it has the same macrolide structure as tacrolimus (Qi et al. 2014). Therefore, ascomycin has been highly valued in various clinical applications (Bugelski et al. 2010; Ianiri et al. 2017; Taieb et al. 2013). In particular, a semisynthetic derivative of ascomycin called pimecrolimus has been used as the first-line treatment for mild-to-moderate atopic dermatitis and plays an important role in the market of immunosuppressive drugs (Bardur et al. 2015; Ho et al. 2003). Due to its complex macrolide structure, ascomycin is difficult to synthesize by chemical methods, and thus is mainly produced by microbiological fermentation (Xin et al. 2015). However, the yield of ascomycin produced via microbiological fermentation is still low and the production costs are high.

Recently, various efforts have been made to improve ascomycin yield through genetic manipulation. For example, the overexpression of some key genes involved in ascomycin biosynthesis, such as *hcd*, *ccr*, *fkbR1*, and *fkbE*, led to a marked increase in ascomycin yield (Song et al. 2017; Wang et al. 2017). In addition, an engineered *S. hygroscopicus* strain with increased chorismatase (FkbO) activity and inactivated pyruvate carboxylase (Pyc), named TD-ΔPyc-FkbO, showed the highest reported ascomycin yield to date, 610.0 mg/L (Qi et al. 2017). Nonetheless, this yield is considered low, as it is not high enough to meet the demands, and the lack of complete genomic information for *S. hygroscopicus* limits further modifications of the strain by genetic manipulation (Wu et al. 2000).

Traditional mutagenesis, and especially some new mutagenesis methods, is an effective and rapid way to enhance the production of secondary metabolites (Sivaramakrishnan and Incharoensakdi 2017; Zhang et al. 2016a). Recently, atmospheric and room temperature plasma (ARTP), which induces mutations at a higher rate than ultraviolet irradiation and *N*-methyl-*N*′-nitro-*N*-nitrosoguanidine mutation, proved to be an efficient tool to generate stable high-yield mutant strains for microorganism breeding (Ottenheim et al. 2018; Ren et al. 2017). ARTP mutagenesis has been combined with diethyl sulfate treatments for the improvement of arachidonic acid production (Li et al. 2015). Additionally, the production of transglutaminase was significantly enhanced in *Streptomyces mobaraensis* by iterative mutagenesis breeding with ARTP (Jiang et al. 2017).

It is necessary to optimize the composition of the culture medium to further enhance ascomycin production. However, in traditional ‘one-factor-at-a-time’ experiments, the effects of various factors can only be investigated one at a time, and this approach fails to evaluate multifactorial interactions among all components, thereby leading to inefficient and time-consuming work (Lee 2018). Response surface methodology (RSM) includes factorial designs and regression analysis for construction of empirical models, making it an excellent statistical tool for increasing the production of valuable metabolites (Chaudhary et al. 2017; Fu et al. 2016). RSM can evaluate all the factors simultaneously and determine the optimal culture conditions for microbes. Compared with traditional optimization, it can save a lot of time when employing RSM to obtain the desirable fermentation medium for ascomycin production.

Herein, we obtained a high-yield *S. hygroscopicus* SFK-36 strain by ARTP-induced mutagenesis and then fermentation medium was optimized by RSM. Finally, scale-up fermentation showed significantly improved ascomycin production by SFK-36.

## Materials and methods

### Strains and primers

*Streptomyces hygroscopicus* ATCC 14891 strain was purchased from ATCC. *S. hygroscopicus* SFK-36 is a mutant of ATCC 14891 strain. Primers used in this study are listed in Additional file 1: Table S1.

### Culture conditions for *S. hygroscopicus*

The *S. hygroscopicus* strain was inoculated onto a slant medium (soluble starch, 10.0 g/L; yeast extract, 4.0 g/L; K_2_HPO_4_, 0.5 g/L; MgSO_4_·7H_2_O, 0.5 g/L; and agar, 20.0 g/L; pH 7.2) at 28 °C for 7 days to harvest spores. The spores were inoculated into 20 mL of the seed medium (corn steep liquor, 8.0 g/L; glucose, 10.0 g/L; cottonseed meal, 3.0 g/L; and KH_2_PO_4_, 1.0 g/L; pH 7.0) in a 250 mL flask incubated at 28 °C and 200 rpm. Then, the 10% (v/v) seed culture was transferred into 250 mL flasks containing 25 mL of the fermentation medium and incubated at 28 °C and 200 rpm. The original fermentation medium (soluble starch, 20.0 g/L; dextrin, 40.0 g/L; yeast powder, 5.0 g/L; peptone, 5.0 g/L; corn steep liquor, 5.0 g/L; K_2_HPO_4_·3H_2_O, 1.0 g/L; (NH_4_)_2_SO_4_, 1.5 g/L; MnSO_4_·H_2_O, 0.5 g/L; MgSO_4_·7H_2_O, 1.0 g/L; CaCO_3_, 1.0 g/L; and soybean oil, 1.0 g/L; pH 6.5) was previously described (Song et al. 2017). Dextrin (dextrose equivalent value 10–15) used in this study was purchased from Shandong Xiwang Co., Ltd., China. Each experiment was repeated three times, and the error bar was used to indicate the standard deviations (SDs).

### ARTP mutagenesis

**Fig. 1:**
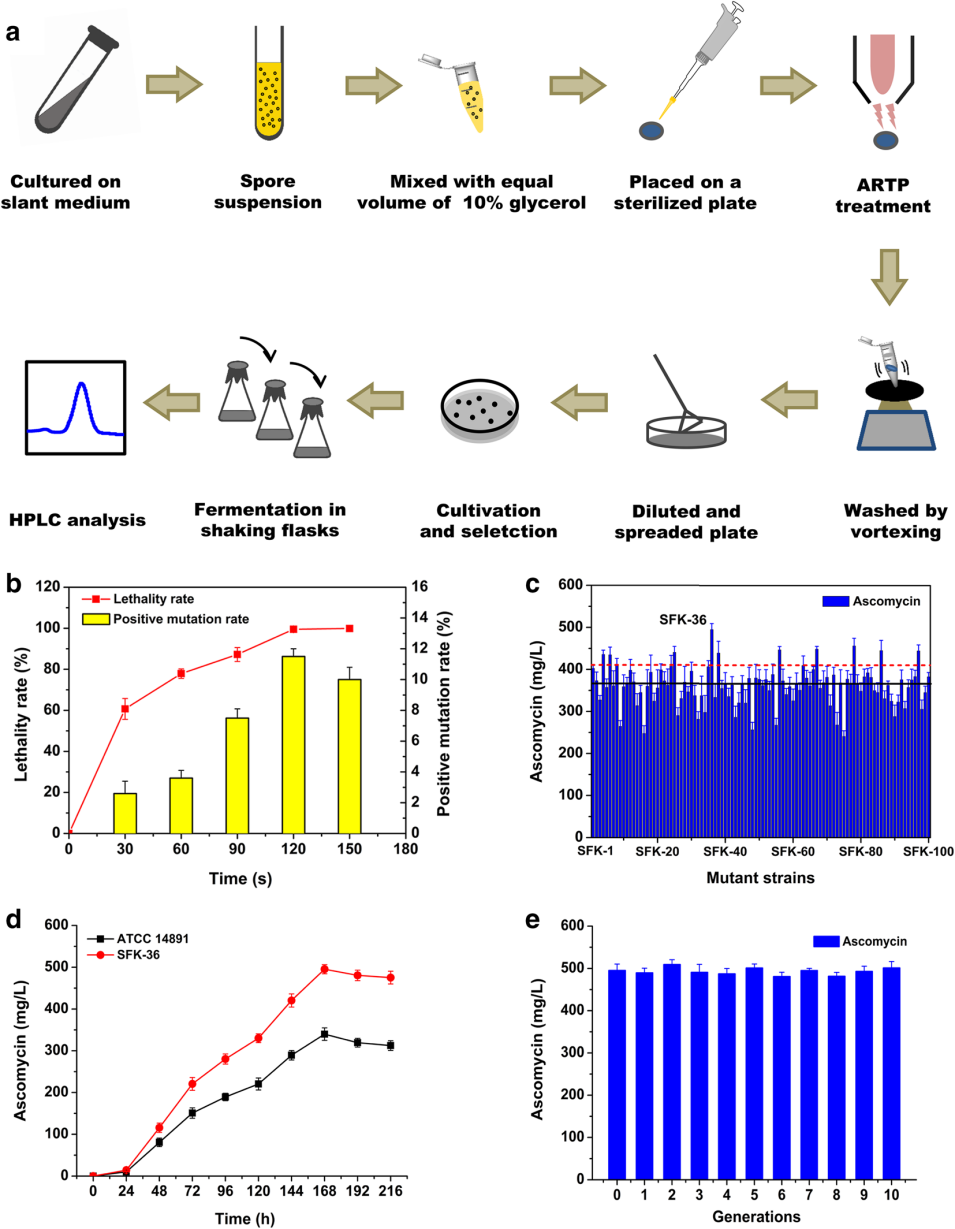
ARTP mutagenesis breeding for *S. hygroscopicus* ATCC 14891 strain. **a** The experimental procedure of high-yield strain screening by ARTP breeding system. **b** Lethality rate and positive mutation rate of ATCC 14891 strain after ARTP mutagenesis. **c** Ascomycin production of the isolated mutants after ARTP mutagenesis (the black solid line represented 373.8 mg/L—the production of original ATCC 14891 strain, while the red dotted line was for the yield of positive mutant strains, in which ascomycin production was found to be over 10% higher relative to ATCC 14891 strain). **d** The comparison of ascomycin production between SFK-36 and ATCC 14891 in shake flask. **e** Genetic stability of SFK-36 in ascomycin production by natural subculture

The original ATCC 14891 strain was treated with ARTP Mutagenesis Breeding Machine (ARTP-M, Wuxi TMAXTREE Biotechnology Co., Ltd., China) following a method reported previously (Ren et al. 2017), with some modifications. As shown in Fig. 1a, the spore suspension was first harvested from a fresh slant medium. Next, 10 μL of the spore solution (10^7^ spores/mL) was placed onto a sterile stainless-steel plate and subjected to plasma irradiation. The working parameters were as follows: radiofrequency power input, 100 W; gas flow of pure helium, 10 L/min; effective distance between the plasma torch nozzle exit and the sample plate, 2 mm; temperature of the plasma jet, room temperature (20–25 °C); and treatment periods for the spores, 0, 30, 60, 90, 120, and 150 s. Afterwards, mutant strains were randomly selected and inoculated into the fermentation medium in shaking flasks.

The lethality rate was calculated as follows:

$${\text{Lethality}}\;{\text{rate}}\;\left( \% \right) = \left[ {\left( {C - S} \right)/C} \right] \times 100\%$$

where, *C* is the total number of colony forming units (CFUs) of the spores without ARTP treatment, and *S* is the CFU number of the mutant strains after ARTP mutagenesis breeding.

In addition, the positive mutation rate was determined as the following equation:

$${\text{Positive}}\;{\text{mutation}}\;{\text{rate}}\;\left( \% \right) = \left( {P/S} \right) \times 100\%$$

where, *P* is the CFU number of mutants with greater than 10% increase in ascomycin production relative to ATCC 14891 strain, and *S* is the CFU number of the mutant strains after ARTP mutagenesis breeding.

### Verification of genetic stability by RAPD analysis

*Streptomyces hygroscopicus* strains were grown in tryptic soy broth medium (TSB; Becton–Dickinson, Sparks, MD, USA) for 2 days at 28 °C and 200 rpm. Culture broth was then centrifuged at 8000×*g* for 5 min to collect mycelium for DNA isolation. The total genomic DNA of *S. hygroscopicus* strains was extracted using DNeasy Blood & Tissue Kit (Qiagen 69504, Germany) according to the manufacturer’s instructions. Randomly amplified polymorphic DNA (RAPD) analysis was carried out with primers RAPD-1, RAPD-2 and RAPD-3 (Additional file 1: Table S1) to map the DNA fingerprint of the SFK-36 mutant strain according to the method reported previously (Martins et al. 2004; Sikora et al. 1997).

### Optimization by RSM and statistical design

The experimental design of RSM was generated in statistical software Design Expert Version 8.0.6. A three-level method of Box–Behnken design included soluble starch (A), peanut meal (B) and soybean oil (C). All variables were set to three levels (− 1, 0, and 1) containing minimum, intermediate, and maximum values according to the preliminary experimental results (Table 1). A total of 17 factorial points were designed and used in the standard order (Table 2). Among these, 12 points represented different combinations of the experimental variables and five additional experimental setups were replications of the central point. Second-order polynomial Eq. (1) was employed to analyze the effects of independent variables on the response as follows (Ju et al. 2015):

1 $$Y = \beta_{0} + \beta_{a} A + \beta_{b} B + \beta_{c} C + \beta_{ab} AB + \beta_{ac} AC + \beta_{bc} BC + \beta_{aa} A^{2} + \beta_{bb} B^{2} + \beta_{cc} C^{2}$$

where, *Y* is the response calculated; *A*, *B* and *C* are the coded values of independent variables; *β*_*0*_, *β*_*a*_, *β*_*b*_, *β*_*c*_, *β*_*ab*_, *β*_*ac*_, *β*_*bc*_, *β*_*aa*_, *β*_*bb*_ and *β*_*cc*_ are the constant regression coefficients of the model.

**Table 1:** Independent variables and different levels used in RSM

Factors (g/L)	Code	Levels of variables		
		− 1	0	+ 1
Soluble starch	A	70.0	80.0	90.0
Peanut meal	B	50.0	60.0	70.0
Soybean oil	C	10.0	15.0	20.0

**Table 2:** Experimental design for media optimization

Run	A	B	C	Ascomycin (mg/L)
1	− 1	0	− 1	852.1
2	− 1	1	0	899.4
3	0	− 1	− 1	954
4	− 1	0	1	1162.6
5	0	0	0	1435.8
6	1	0	− 1	1054.9
7	0	0	0	1397.5
8	1	0	1	1123.1
9	0	1	− 1	703.5
10	1	1	0	758.2
11	1	− 1	0	1228.4
12	0	− 1	1	1094.4
13	0	0	0	1416.2
14	0	1	1	855.1
15	0	0	0	1458.2
16	0	0	0	1408.8
17	− 1	− 1	0	1089.8

### Culture of *S. hygroscopicus* in a 5 L fermenter

The *S. hygroscopicus* SFK-36 strain was cultured in 750 mL flasks containing 50 mL of the seed medium at 28 °C and 200 rpm. Next, the seed culture was inoculated (10%, v/v) into the 5 L fermenter containing 2.5 L of the fermentation medium. Fermentation was carried out at 28 °C for 192 h with aeration at 1.0 vvm (air volume/culture volume/min). The agitation rate was set automatically to maintain dissolved oxygen levels higher than 20% during the whole fermentation process, and pH was maintained at 6.5 using 1 mol/L ammonia and 2 mol/L H_2_SO_4_ starting from 72 h of fermentation.

### Analysis of transcriptional levels by quantitative RT-PCR

Fermentation culture broth (10 mL) of the *S. hygroscopicus* ATCC 14891 and SFK-36 were centrifuged at 8000×*g* for 5 min to collect mycelium cultured for 3 days and 6 days, respectively. Then, the total RNA was obtained using the Ultrapure RNA Kit (CWBIO, Beijing, China) according to the manufacturer’s instructions. The residual genomic DNA in the RNA sample was removed by RNase-free DNase I (Takara, Dalian, China). To verify elimination of the residual DNA, the primers 16S-RT-F/16S-RT-R (Additional file 1: Table S1) were used to amplify the total RNA. Reverse transcription-PCR (RT-PCR) analysis was performed with the PrimeScript™ RT Reagent Kit (Takara, Dalian, China) according to the instructions provided by the manufacturer.

To determine the transcription units in ascomycin biosynthetic gene cluster, 10 pairs of primers (Additional file 1: Table S1) were used to amplify the cDNA of *S. hygroscopicus* ATCC 14891 strain, including *fkbW*-*U*-F/*fkbW*-*U*-R, *fkbU*-*R2*-F/*fkbU*-*R2*-R, *fkbR2*-*R1*-F/*fkbR2*-*R1*-R, *fkbF*-*G*-F/*fkbF*-*G*-R, *fkbH*-*I*-F/*fkbH*-*I*-R, *fkbK*-*L*-F/*fkbK*-*L*-R, *fkbL*-*C*-F/*fkbL*-*C*-R, *fkbN*-*Q*-F/*fkbN*-*Q*-R, *fkbP*-*A*-F/*fkbP*-*A*-R, *fkbC*-*B*-F/*fkbC*-*B*-R. The corresponding PCR products were individually identified by sequencing.

To analyze the transcriptional levels in *S. hygroscopicus* ATCC 14891 and SFK-36, quantitative real-time polymerase chain reaction (qRT-PCR) was conducted with TB Green™ Premix Ex Taq™ II (Takara, Dalian, China) according to the manufacturer’s instructions. Seven primer pairs were used to determine the transcriptional levels of ascomycin biosynthetic gene cluster, including *fkbW*-RT-F/*fkbW*-RT-R, *fkbU*-RT-F/*fkbU*-RT-R, *fkbR1*-RT-F/*fkbR1*-RT-R, *fkbE*-RT-F/*fkbE*-RT-R, *fkbB*-RT-F/*fkbB*-RT-R, *fkbO*-RT-F/*fkbO*-RT-R, *fkbS*-RT-F/*fkbS*-RT-R, 16S-RT-F/16S-RT-R (Additional file 1: Table S1). All the amplicons were confirmed by sequencing. The transcriptional levels were normalized using gene 16S rRNA as the internal control (Wang et al. 2018). The fold changes of test genes were quantified using 2^−ΔΔCt^ method (Livak and Schmittgen 2001). Each qRT-PCR experiment was performed for three times, and the error bar was used to show the standard deviations (SDs).

### Analytical methods

To extract ascomycin from the fermentation broth sample, the cultures were diluted with four volumes of acetone and subjected to ultrasonication (50 Hz) for 20 min and then centrifuged at 13,780×*g* for 3 min. After centrifugation, the supernatant was filtered through a 0.22 μm filter and then analyzed by high-performance liquid chromatography (HPLC 1260 instrument, Agilent, USA) on a Hypersil BDS C_18_ column (5 μm, 4.6 × 150 mm) with monitoring at 210 nm. The mobile phase was deionized water with acetonitrile (35:65, v/v) at a flow rate of 1.0 mL/min at 55 °C. Ascomycin standard (Bioaustralis Fine Chemicals) was used as a control to make standard curves for quantitative analysis.

The biomass of each fermentation sample was determined as packed mycelium volume (PMV). Briefly, culture broth (1.5 mL) was centrifuged at 13,780×*g* for 5 min to measure PMV. The total residual sugar in the culture broth was determined by a concentrated sulfuric-acid method as previously reported (Du et al. 2017).

## Results

### ARTP mutagenesis and strain screening

To determine the optimal treatment time for ARTP mutagenesis, the lethality and positive mutation rates of different treatment time (0, 30, 60, 90, 120, and 150 s) were determined. When ATCC 14891 strain was treated with the plasma for 120 s, the lethality rate of ARTP mutagenesis reached 99.5% and the highest positive mutation rate of 11.6% was obtained (Fig. 1b). Therefore, the optimal operating time of ARTP breeding for ATCC 14891 strain was set to 120 s. Finally, 100 mutant strains were selected for the assessment of ascomycin production in flask culture. As presented in Fig. 1c, a total of 12 positive mutants were obtained. Of these, SFK-36 showed the highest ascomycin yield (495.3 mg/L), a 32.5% increase as compared with the original strain (373.8 mg/L). The time course of ascomycin production clearly revealed that SFK-36 produced more ascomycin than ATCC 14891 strain (Fig. 1d). The significant increase in ascomycin yield confirmed that ARTP mutation breeding is an efficient method for generating high-producing mutants.

The genetic stability of the SFK-36 mutant strain was investigated by subculturing for 10 generations. In each generation, the strain of each generation was transferred into fresh seed medium and then inoculated into fermentation medium in shake flasks for 7 days. The results showed that ascomycin yield of these generations ranged from 478.2 to 509.1 mg/L (Fig. 1e), indicating no significant difference. In particular, a highly similar DNA fingerprint was observed after 10 generations (Additional file 1: Figure S2), thus, indicating that SFK-36 is a genetically stable mutant strain that can be used for ascomycin high-yield production.

### Transcriptional analysis of ascomycin biosynthetic gene cluster

**Fig. 2:**
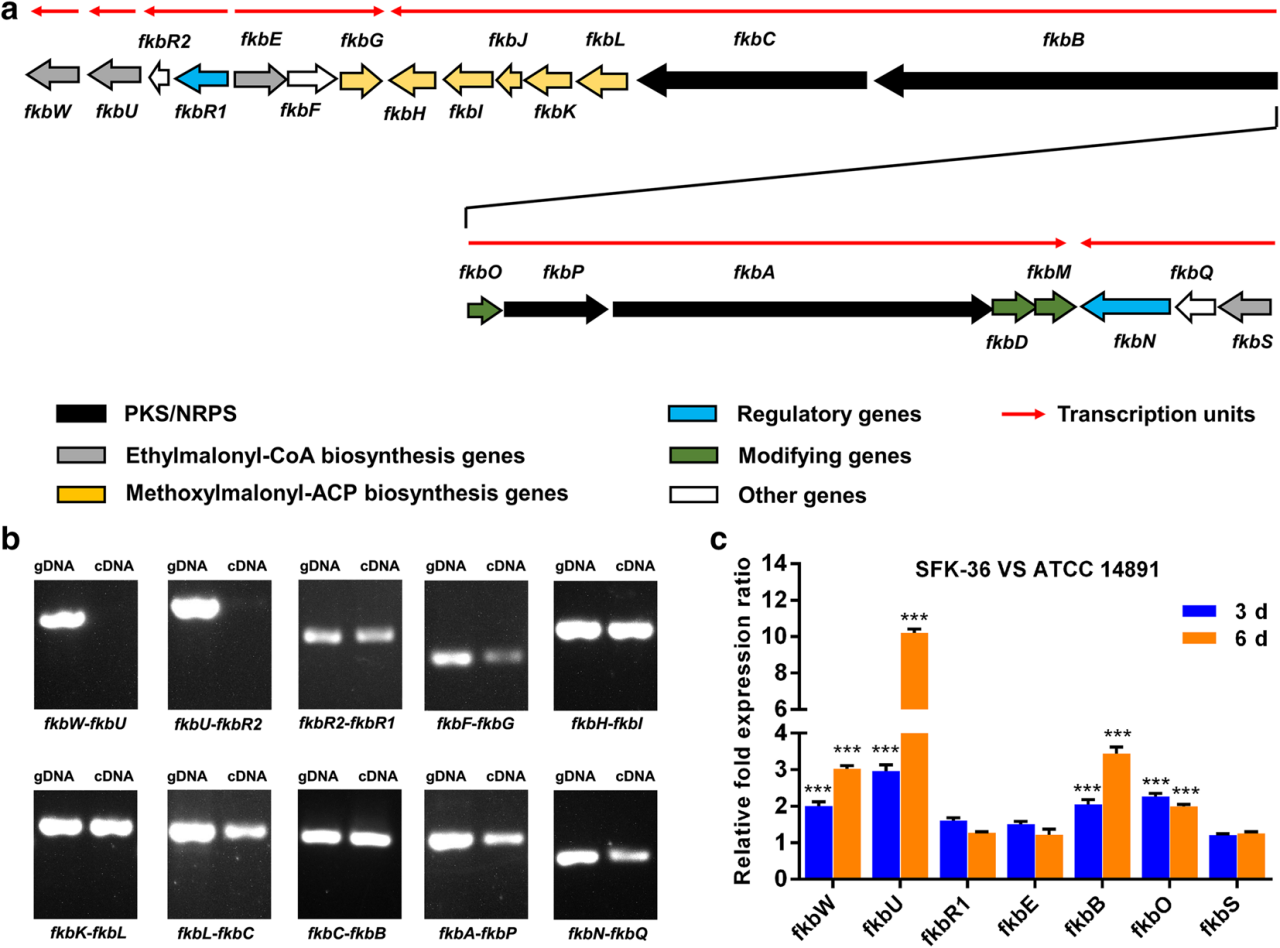
Transcriptional analysis of ascomycin biosynthetic gene cluster in *S. hygroscopicus*. **a** Genetic organization of co-transcription units in ascomycin biosynthetic gene cluster. **b** Co-transcriptional analysis of the ascomycin biosynthetic gene cluster by RT-PCR. Genomic DNA (gDNA) and cDNA of *S. hygroscopicus* 14891 strain were used for PCR amplification. **c** Relative expression levels of ascomycin biosynthetic gene cluster in *S. hygroscopicus* SFK-36, compared with those in ATCC 14891 strain. Totally, seven genes were selected to indicate the expression level of co-transcription units (***P < 0.001)

To understand the possible causes leading to production improvement, the transcriptional analysis of ascomycin biosynthetic gene cluster was investigated. Firstly, RT-PCR was applied to determine co-transcription units in the gene cluster by amplifying the cDNA of *S. hygroscopicus* ATCC 14891 strain with primers (Additional file 1: Table S1). The genomic DNA of *S. hygroscopicus* ATCC 14891 strain was used as the control. As shown in Fig. 2a, b, there are totally seven co-transcription units in the ascomycin biosynthetic gene cluster, including *fkbW*, *fkbU*, *fkbR1/R2*, *fkbE/F/G*, *fkbB/C/L/K/J/I/H*, *fkbO/P/A/D/M*, and *fkbS/Q/N*.

To compare the relative expression levels of the ascomycin biosynthetic gene cluster in *S. hygroscopicus* SFK-36 and ATCC 14891 strain, qRT-PCR was then performed to analyze the samples collected at 3 days and 6 days, respectively. One gene in each of the seven co-transcription units (*fkbW*, *fkbU*, *fkbR1*, *fkbE*, *fkbB*, *fkbO*, and *fkbS*) was selected to determine the corresponding expression levels. The results showed that the expression levels of four co-transcription units, *fkbW*, *fkbU*, *fkbB/C/L/K/J/I/H* and *fkbO/P/A/D/M*, were at least twofold higher in *S. hygroscopicus* SFK-36 than in the ATCC 14891 strain (Fig. 2c). The upregulation of gene products included in these co-transcription units might lead to the improvement of ascomycin production in SFK-36 mutant strain.

### The influence of carbon sources on ascomycin production

**Fig. 3:**
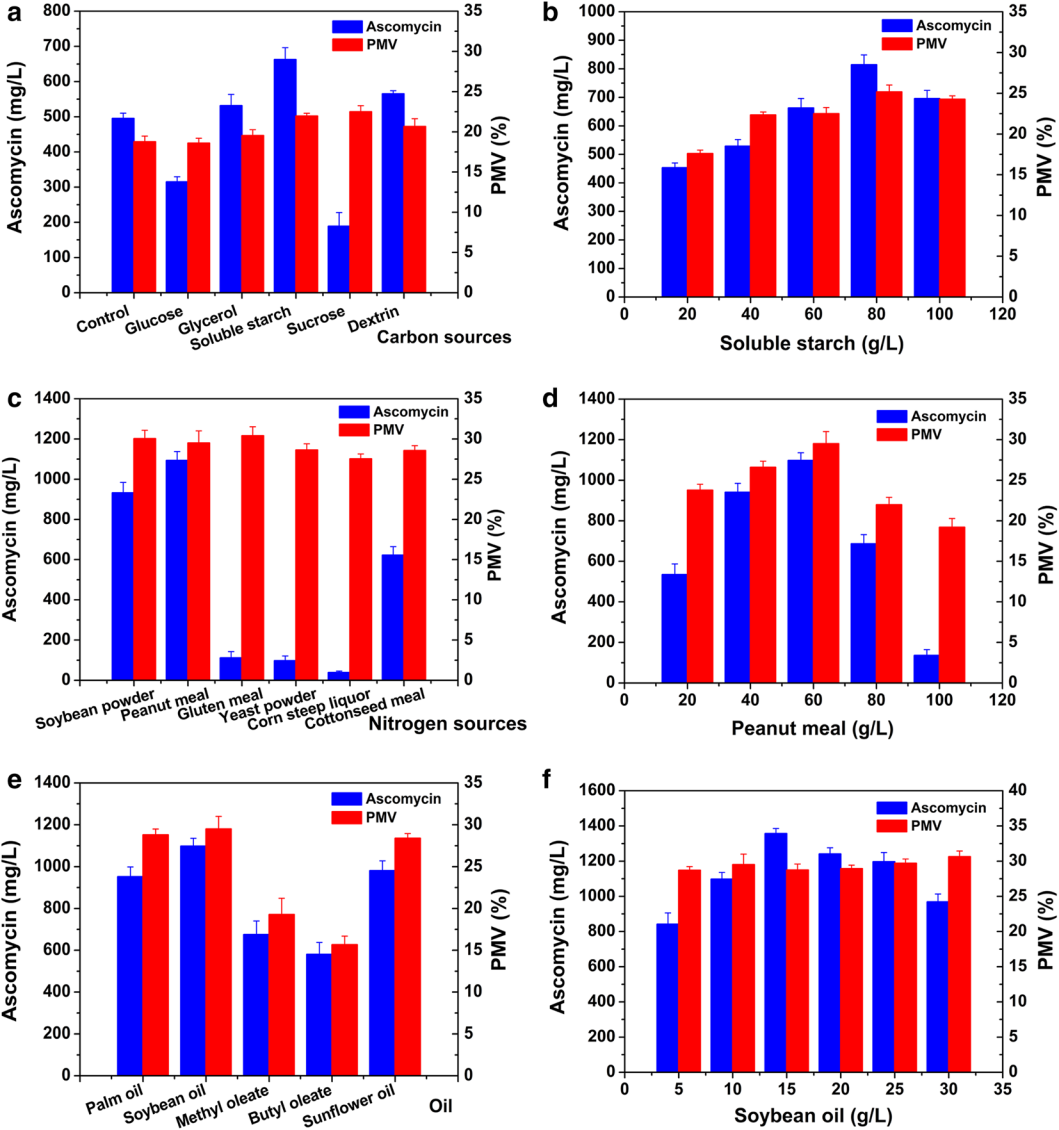
Effect of different fermentation media on ascomycin production by *S. hygroscopicus* SKF-36. Effect of different carbon sources (**a**), soluble starch concentrations (**b**), nitrogen sources (**c**), peanut meal concentrations (**d**), oil (**e**), and soybean oil concentrations (**f**) on ascomycin production and biomass of SKF-36

To further improve ascomycin production, the effects of five carbon sources on ascomycin production were evaluated individually by replacing the carbon source (20 g/L starch and 40 g/L dextrin) in original medium (Song et al. 2017). The selected carbon source included glucose, glycerol, soluble starch, sucrose, and dextrin (all at a concentration of 60 g/L). The original medium was served as a control. As depicted in Fig. 3a, the biomass of SFK-36 after culture in medium with different carbon sources, ranged from 18.6 to 22.5% (PMV), indicating no significant difference in mycelium growth. Nevertheless, in the medium containing starch, dextrin or glycerol, ascomycin production were all higher relative to the control, and the highest ascomycin yield of 662.7 mg/L was obtained in the soluble starch medium, which is 33.8% higher than that in the original medium (495.3 mg/L). In contrast, glucose and sucrose exerted a negative effect on ascomycin production, and reduced the yield to 312.9 and 188.8 mg/L, respectively. Next, the effects of different soluble starch concentrations on ascomycin production were studied (Fig. 3b). The results revealed that the yield and biomass increased with the soluble starch concentration, from 20 to 80 g/L, and the highest yield was 814.4 mg/L.

### The influence of nitrogen sources on ascomycin production

To determine the optimal nitrogen sources for ascomycin production, six commonly used nitrogen sources, soybean powder, peanut meal, gluten meal, yeast powder, corn steep liquor and cottonseed meal were tested at 60 g/L. As shown in Fig. 3c, biomass in the culture media with different starch sources ranged from 27.5 to 30.3%, indicating that all the tested nitrogen sources can effectively support cell growth. The highest ascomycin yield (1093.3 mg/L) was obtained in the fermentation medium containing peanut meal. This yield is 120.7% higher than that in the original medium. In contrast, the addition of yeast powder, gluten meal, and corn steep liquor inhibited ascomycin production, and the yields in medium containing these starch sources were less than 200 mg/L. Then, ascomycin production was assessed at different concentrations of peanut meal. As presented in Fig. 3d, both ascomycin production and biomass increased when peanut meal concentration was increased from 20 to 60 g/L. Therefore, the highest yield of 814.4 mg/L was observed in medium containing 60 g/L peanut meal.

### The influence of oil on ascomycin production

Oil is rich in fatty acids, which can be degraded into acyl-coenzymes A by microbes. Acyl-coenzymes A are recognized as important precursors for the biosynthesis of secondary metabolites in *Streptomyces* (Chen et al. 2012; Duan et al. 2012; Lu et al. 2016). As shown in Fig. 3e, the five tested oils exerted different actions on ascomycin production. The SFK-36 strain in the soybean oil medium manifested the highest ascomycin yield (1097.9 mg/L) and biomass (29.5%). In contrast, both methyl oleate and butyl oleate decreased the ascomycin yield and biomass. To further improve ascomycin production, different concentrations of soybean oil were added to the fermentation medium (Fig. 3f). The corresponding biomass ranged from 28.7 to 30.7%. Ascomycin yield increased with the addition of soybean oil, but decreased when the concentration of soybean oil was higher than 15 g/L, and the highest ascomycin yield was 1356.7 mg/L.

### Parameter optimization by RSM for ascomycin production

Three factors including soluble starch (A), peanut meal (B) and soybean oil (C) were considered at three different levels. A total of 17 experimental points were obtained, and the corresponding results are listed in Table 2. Finally, multiple regression analysis generated the following equation for modelling ascomycin production (R^2^ = 0.99 and *P *< 0.001):

2 $$Y = 1423.30 + 20.09A - 143.80B + 83.84C - 68.95AB - 60.58AC + 2.80BC - 141.46A^{2} - 287.89B^{2} - 233.66C^{2}$$

Results of analysis of variance in this model are given in Table 3 and were employed to estimate the model implications. The *F*-value was determined next to evaluate the mean square regression and residual of the predictive model. The model *F*-value of 74.75 indicated that the model was statistically significant. The *F*-value of lack-of-fit testing was 4.65, which implied that the lack-of-fit was not significantly associated with pure error and that the model well fitted the experimental data. Additionally, the *P*-value was calculated to determine the significance of the terms and interactions between experimental variables. Analysis revealed that *B*, *C*, *AB*, *AC*, *A*^*2*^, *B*^*2*^, and *C*^*2*^ were statistically significant model terms (*P *< 0.05), indicating that ascomycin production can be analyzed and estimated by means of the model.

**Table 3:** ANOVA for response surface quadratic model

Source	Sum of square	Degrees of freedom	Mean square	*F*-value	*P*-value Prob > F	Significant
Model	99,2600.00	9	110,300.00	74.75	< 0.0001	***
A	3228.06	1	3228.06	2.19	0.1826	
B	165400.00	1	165400.00	112.13	< 0.0001	***
C	56229.81	1	56229.81	38.11	0.0005	***
AB	19572.01	1	19572.01	13.28	0.0083	**
AC	14677.32	1	14677.32	9.95	0.0161	*
BC	31.36	1	31.36	0.021	0.8882	
A^2^	84259.53	1	84259.53	57.11	0.0001	***
B^2^	349000.00	1	349000.00	236.54	< 0.0001	***
C^2^	229900.00	1	229900.00	155.82	< 0.0001	***
Residual	10327.10	7	1475.30			
Lack of Fit	8026.54	3	2675.51	4.65	0.0858	
Pure error	2300.56	4	575.14			
Cor Total	1003000.00	16				

* P < 0.05; **P < 0.01; ***P < 0.001; C.V. % = 3.46; R^2^ = 0.99; Adj R^2^ = 0.98; Pred R^2^ = 0.87

**Fig. 4:**
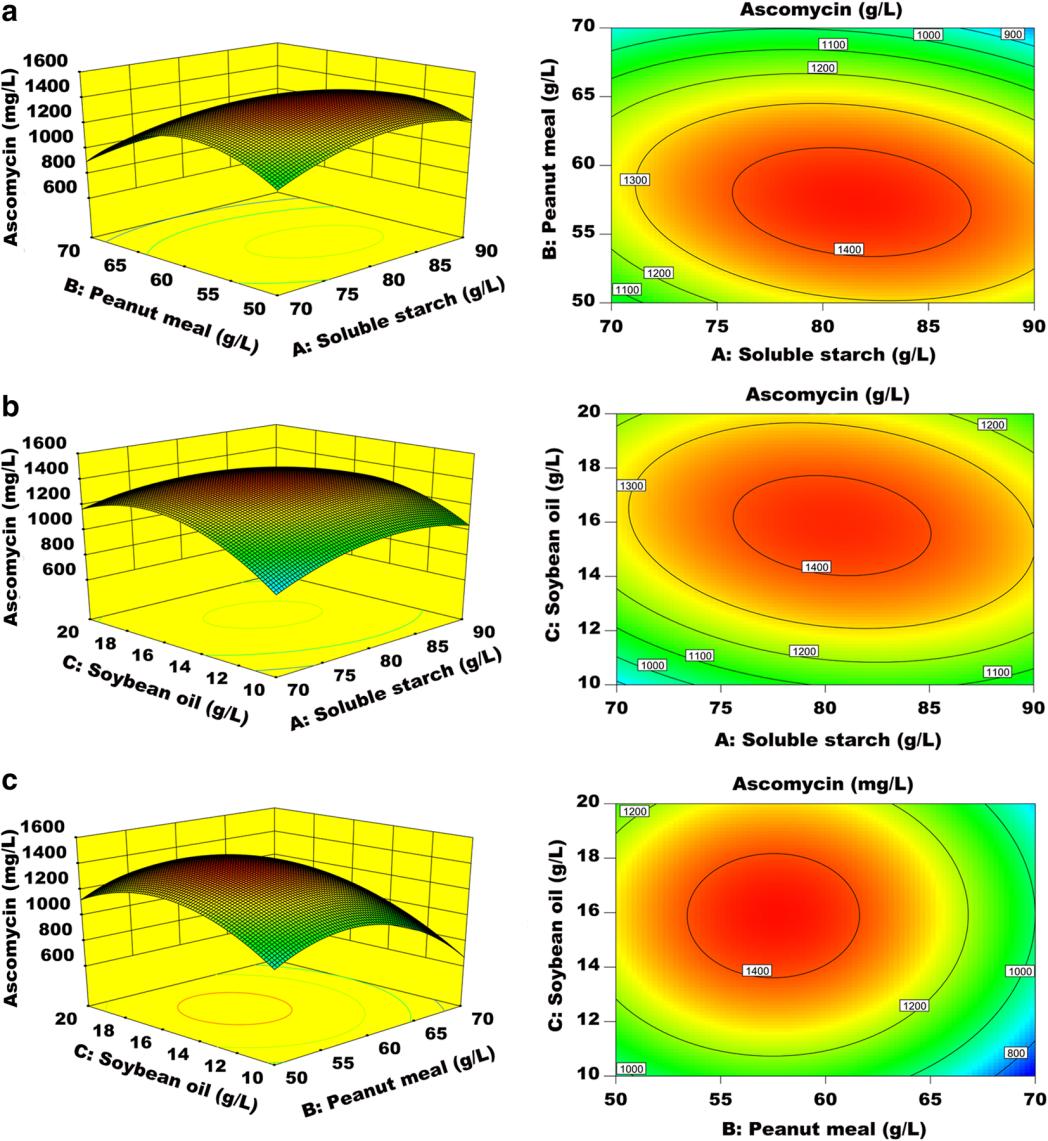
The effect of mutual interactions among soluble starch,peanut meal and soybean oil on ascomycin production by *S. hygroscopicus* SKF-36. Response surfaces and contour plots of effects of interactions between soluble starch and peanut meal (**a**), soluble starch and soybean oil (**b**), peanut meal and soybean oil (**c**) on ascomycin production by SKF-36

The three-dimensional (3D) response surface plots and two-dimensional (2D) contour plots representing the predictive equation are shown in Fig. 4. The 3D response surface plots were expected to form a convex shape, and the peak of the plot represents the optimum combination of two tested factors. The 2D contour plots had an oval shape when one factor was fixed, indicating the significant interaction between the two variables. Therefore, the optimal combination predicted by the 3D response surface plots and 2D contour plots was 81.0 g/L soluble starch, 57.4 g/L peanut meal, and 15.8 g/L soybean oil.

### Validation of the predictive model

**Fig. 5:**
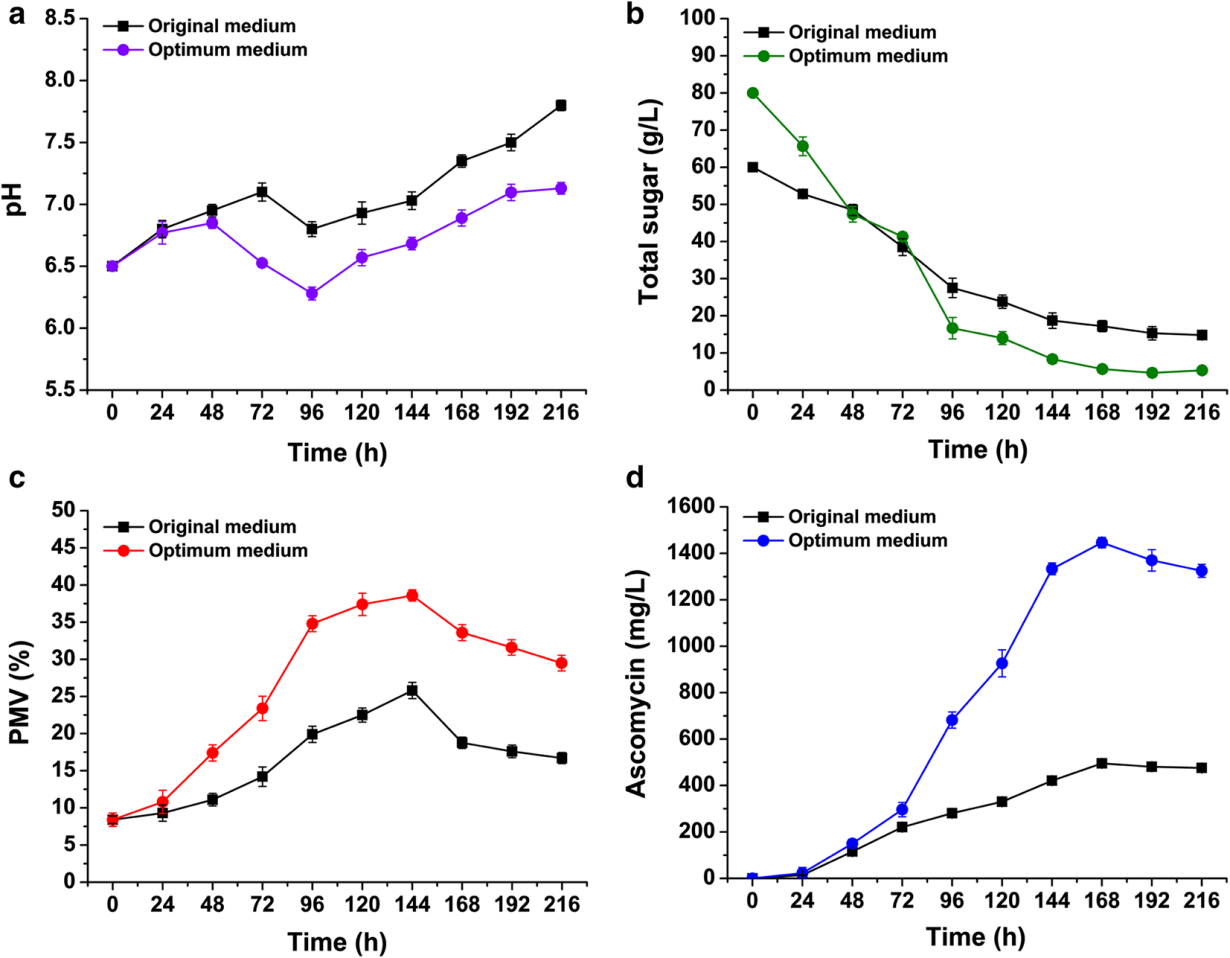
Comparison of fermentation process between optimized medium and original medium by *S. hygroscopicus* SFK-36 in flask culture. Changes of pH (**a**), consumption of carbon source (**b**), time course of growth (**c**), and ascomycin accumulation (**d**) of SFK-36 in optimized medium and original medium

The optimum combination of the experimental variables (A, B, and C) was predicted via response surface and contour plots. The optimal fermentation medium was found to contain the following components (g/L): soluble starch, 81.0; peanut meal, 57.4; soybean oil, 15.8; MnSO_4_·H_2_O, 0.5; K_2_HPO_4_·3H_2_O, 1.0; MgSO_4_·7H_2_O, 1.0; and CaCO_3_, 1.0. SFK-36 was cultured in the optimized medium and original medium separately to evaluate ascomycin production (Fig. 5). In the optimized medium, pH was in the range of 6.0–7.0, which turned out to be lower than that in the original medium during the whole fermentation process (Fig. 5a). Additionally, SFK-36 showed a faster carbon utilization rate in the optimized medium, as the final residual sugar concentrations at the end of fermentation in the optimized and original mediums were 5.2 and 15.1 g/L, respectively (Fig. 5b). The biomass of SFK-36 was 38.5% and 25.8% in the optimized medium and original medium, respectively, at 144 h (Fig. 5c), suggesting that the optimized medium was more suitable for the growth of SFK-36 mycelia. Ascomycin yield reached 1446.3 mg/L at 168 h in the optimized medium (Fig. 5d); this figure was close to the predicted value of 1450.0 mg/L (99.7%).

### Effects of fermentation conditions on ascomycin production

**Fig. 6:**
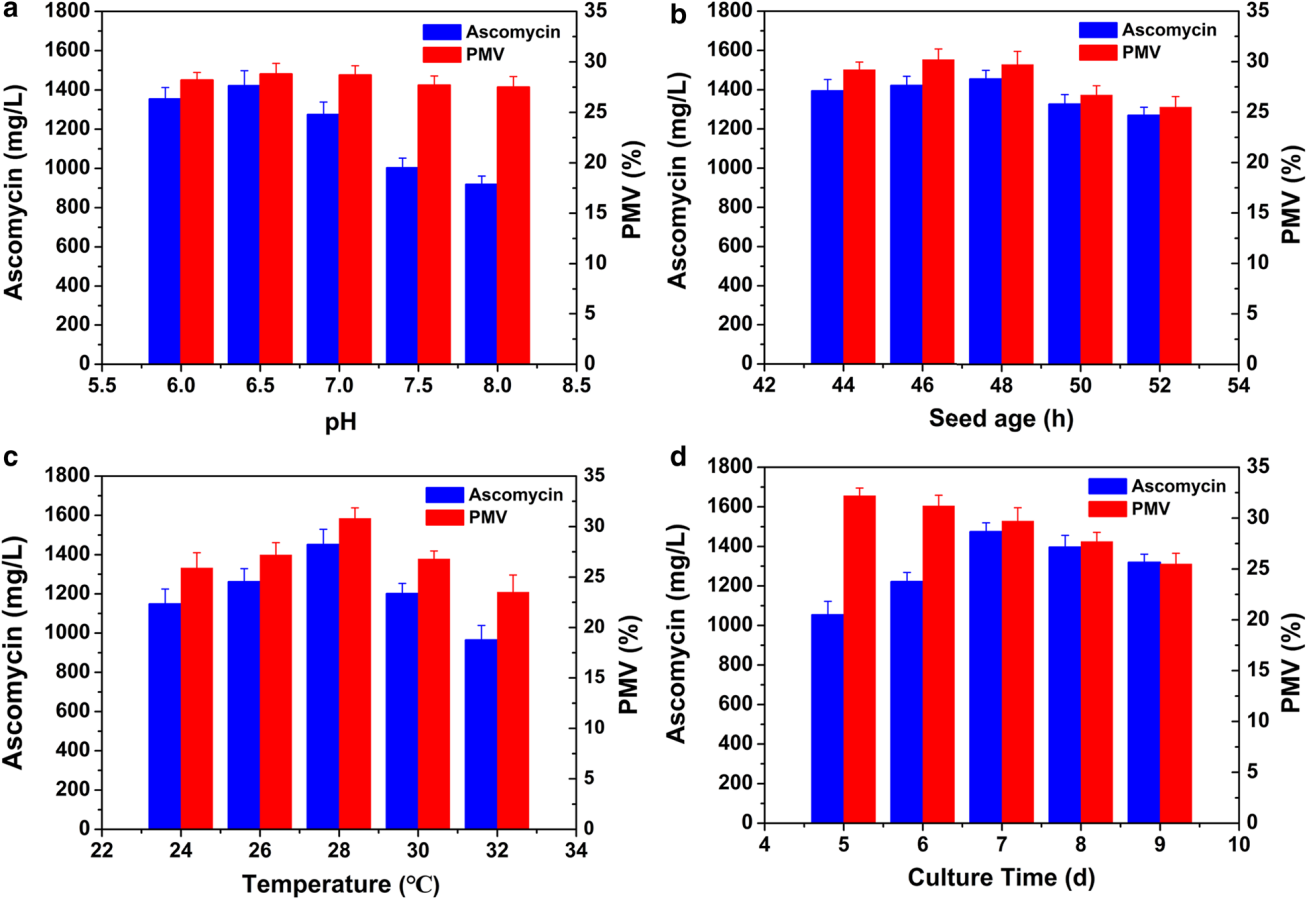
Optimization of fermentation conditions for ascomycin production by *S. hygroscopicus* SFK-36 in shake flasks. Effect of pH (**a**), seed age (**b**), temperature (**c**), culture time (**d**) on ascomycin production and biomass of SFK-36

The influence of pH on production is presented in Fig. 6a. Mycelium growth showed no significant difference when pH was varied from 6.0 to 6.5. Ascomycin yield reached 1421.4 mg/L at pH 6.5. As presented in Fig. 6b, SFK-36 produced relatively high concentrations of ascomycin when the seed age was in the range of 44–48 h. The effect of temperature on ascomycin production is shown in Fig. 6c. Both ascomycin yield and biomass increased when the temperature was increased from 24 to 28 °C, suggesting that higher temperatures promoted mycelium growth and ascomycin accumulation. The highest ascomycin yield of 1451.4 mg/L was obtained at 28 °C. Nonetheless, it exerted a negative effect on ascomycin production and mycelium growth when temperature was higher than 28 °C. Additionally, culture time was an important factor influencing mycelium growth and ascomycin accumulation of SFK-36. The effects of different culture time were studied in the optimized medium (Fig. 6d). The highest ascomycin yield was obtained when SFK-36 was cultured in the optimized medium for 7 days, reaching 1468.2 mg/L.

### Scale-up fermentation in a 5 L bioreactor

**Fig. 7:**
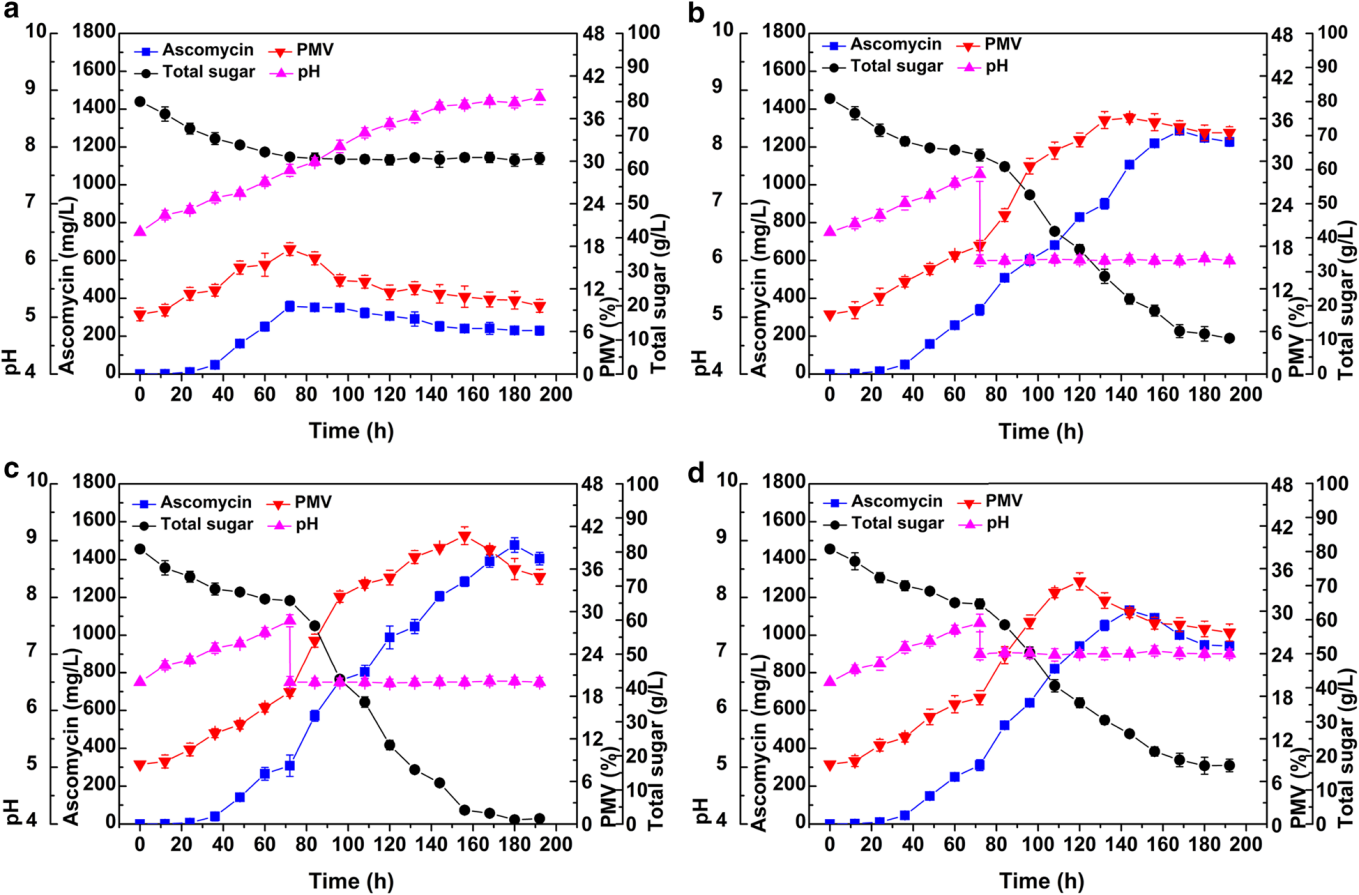
Scale-up fermentation of *S. hygroscopicus* SFK-36 under optimized fermentation conditions in a 5 L fermenter. The pH-controlling strategies were conducted with no controlling (**a**), or maintained at a constant pH of 6.0 (**b**), 6.5 (**c**), 7.0 (**d**), respectively

Scale-up fermentation of ascomycin by SFK-36 in the optimized medium was carried out in a 5 L fermenter. The time course of the fermentation process is depicted in Fig. 7a. During the early stage of fermentation (0–36 h), total sugar in the fermentation broth rapidly decreased from 80 to 68.1 g/L, while biomass increased gradually to 11.8%. SFK-36 began to produce ascomycin at 36 h. The highest ascomycin concentration (357.9 mg/L) and biomass (17.6%) were observed at 72 h. Afterwards, both ascomycin production and biomass gradually decreased until the end of fermentation. SFK-36 stopped consuming the carbon source in the fermentation broth after 72 h, and total sugar remained relatively constant at 63.5 mg/L. Compared to flask culture, pH in the 5 L bioreactor increased from 6.5 to 8.9 throughout the whole fermentation period. Soluble starch was not utilized after 72 h, indicating that SFK-36 strain cannot efficiently utilize this carbon source when pH is higher than 7.5.

Therefore, a pH control strategy for the fermentation process was developed to enhance ascomycin production. To determine the optimal control point, the pH was maintained at three levels (6.0, 6.5 and 7.0) starting at 72 h. When pH was maintained at 6.0 (Fig. 7b), total sugar began decreasing stably following an increase in biomass and ascomycin production. On the other hand, abundant residual sugar (10.5 g/L) was observed at the end of fermentation, indicating that the carbon source cannot be completely utilized at pH 6.0. The highest ascomycin yield of 1285.8 mg/L was reached at 168 h. Similar results were observed when pH was maintained at 6.5 (Fig. 7c), except that the carbon source was consumed completely at the end of the fermentation period. Ascomycin yield increased to 1476.9 mg/L. As illustrated in Fig. 7d, when the medium was maintained at pH 7.0, concentration of the carbon source remained high (17.2 g/L), and ascomycin production diminished to 1129.4 mg/L. Therefore, pH control was an effective method for improving ascomycin production in the fermenter.

## Discussion

The ARTP mutagenesis can cause greater DNA damage with higher diversity than UV irradiation or chemical mutagens, because of the reactive chemical species produced by the helium-based ARTP system (Ottenheim et al. 2018). DNA structure can be disrupted by these active chemicals, thereby inducing the microbial SOS repair system, which has a high error tolerance rate (Bugay et al. 2015; Zhang et al. 2015). Therefore, a variety of mismatches will be generated in the repair process, resulting in a large number of mutants. Accordingly, the positive mutant rate increased to 11.6%, making it easier to obtain stable high-yield strains. ARTP has been reported as an efficient method for improvement of secondary metabolites in *Streptomyces* (Ottenheim et al. 2018). In addition, ARTP mutagenesis substantially improved the yield of arachidonic acid produced by *Mortierella alpina* (Li et al. 2015). However, the development of ascomycin-producing strains by ARTP mutagenesis has not been reported so far. Recently, some efforts have been made to improve ascomycin production (Table 4). Few studies employed traditional mutation breeding methods, such as titanium sapphire laser mutagenesis and shikimic acid enduring screening, to obtain two stable high-yield strains, FS35 and SA68, respectively (Qi et al. 2012, 2014). These two strains were then genetically engineered to enhance ascomycin production. For example, ascomycin production was increased to 438.9 mg/L by overexpression of *hcd* and *ccr* in FS35 (Wang et al. 2017). Song et al. overexpressed the gene encoding the regulatory protein FkbR1 and its target gene *fkbE* in FS35 strain (Song et al. 2017). Additionally, engineered strain TD-ΔPyc-FkbO was constructed from SA68, thus improving ascomycin production to 610.0 mg/L (Qi et al. 2017). Nevertheless, ascomycin yield in these studies was still low to satisfy industrial demand. Compared with the above *S. hygroscopicus* strains, the SFK-36 mutant strain obtained via ARTP mutagenesis was found to have higher ascomycin yield, suggesting that ARTP mutagenesis is an effective way to enhance ascomycin production in *S. hygroscopicus*.

**Table 4:** Ascomycin production by different *S. hygroscopicus* strains

Strains (*S. hygroscopicus*)	Methods	Scale	Ascomycin (mg/L)	References
FS35	Titanium sapphire laser system^a^	1 L flask	305.6	Qi et al. (2012)
SA68	Titanium sapphire laser system and shikimic acid enduring screening model^b^	1 L flask	450.0	Qi et al. (2014)
HA-Hcd-Ccr	Overexpression of *hcd* and *ccr*^c^	1 L flask	438.9	Wang et al. (2017)
OfkbRE	Overexpression of *fkbR1* and *fkbE*^d^	1 L flask	536.7	Song et al. (2017)
TD-ΔPyc-FkbO	Overexpressing *fkbO* and inactivating *Pyc*^e^	3 L fermenter	610.0	Qi et al. (2017)
SFK-36	ARTP mutagenesis and medium optimization	5 L fermenter	1476.9	This study

^a^The FS35 mutant strain was obtained in the optimal irradiation conditions (25 mW for 6 min) by the Titanium sapphire laser system (810 nm, 76 MHz, 150 fs)

^b^Titanium sapphire laser system (25 mW for 6 min) was combined with an enduring selection mode containing 5 g/L shikimic acid to generate SA68 mutant strain

^c^Functional gene *hcd* and *ccr* were overexpressed together under the control of promoter PermE* and integrated into ΦC31 integration site of FS35 strain to generate engineering strain HA-Hcd-Ccr

^d^Positive regulatory gene *fkbR1* and functional gene *fkbE* were overexpressed together under the control of PermE* and integrated into ΦC31 integration site of FS35 strain

^e^Gene deletion plasmid pΔPyc derived from pKC1139 was used for inactivating *Pyc* gene in SA68 strain to generate engineering strain TD-ΔPyc. And then, *fkbO* gene under the control of PermE* was integrated into ΦC31 integration site of strain TD-ΔPyc through conjugal transfer

The genomes of *Streptomyces* sp. are characterized by extensive polycistronic organizations (Wei et al. 2018; Zhang et al. 2016b), thus, the whole ascomycin biosynthetic gene cluster was divided into seven co-transcription units, and the transcription of these units was analyzed by RT-PCR. The transcriptional levels of several genes, including *fkbW*/*U*/*B*/*C*/*L*/*K*/*J*/*I*/*H*/*O*/*P*/*A*/*D*/*M*, were significantly upregulated. It has been reported that proteins encoded by *fkbW* and *fkbU*, participate in the synthesis of ethylmalonyl-CoA, a precursor of ascomycin biosynthesis (Wang et al. 2017). The polyketide synthase (PKS) module consisting of *fkbB*, *fkbC* and *fkbA*, plays an important role in the synthesis of ascomycin skeleton (Wu et al. 2000). Additionally, post-modification of ascomycin structure depends on the modifying enzymes encoded by *fkbO*, *fkbD* and *fkbM* (Qi et al. 2017; Wu et al. 2000). The upregulated transcription of these functional genes could partly explain the improvement in ascomycin production observed in the SFK-36 mutant strain.

Both mycelium growth and metabolite accumulation are affected by the composition of the fermentation medium (Chen et al. 2015; Huang et al. 2018). In single-factor experiments, we found that starch and oil as carbon sources and peanut meal as a nitrogen source can stably provide nutrients and energy for microbial growth and ascomycin production. In contrast, glucose and sucrose exerted a negative effect on ascomycin production in this study, which might be explained as that *S. hygroscopicus* strain could rapidly utilize the glucose and sucrose for growth at the early stage, therefore decreasing the accumulation of secondary metabolites (Deutscher 2008). Similar repressive effect of glucose and glycerol on tacrolimus was observed in *S. tsukubaensis* (Ordóñez-Robles et al. 2017). To investigate the interactions among different components of the fermentation medium and to obtain multiple responses at the same time, RSM was applied to design experiments and to identify optimum conditions with fewer experimental trials. Furthermore, significant interactions among the factors could be identified (Table 3). The results revealed that the interaction between soluble starch and peanut meal was statistically significant (*P *= 0.0083). In addition, a statistically significant interaction was also observed between soluble starch and soybean oil (*P *= 0.0161).

In our study, a high-producing *S. hygroscopicus* SFK-36 mutant strain was obtained by ARTP mutagenesis, and ascomycin production was markedly improved in the optimum fermentation medium designed by RSM. To the best of our knowledge, our methods generated the highest ascomycin yield reported so far. This finding means that the combination of conventional mutagenesis and rational medium optimization is an effective approach for improving ascomycin production.

## Additional file

**Additional file 1: Table S1.** Sequences of primer pairs used in this study. **Figure S1.** Structure of ascomycin and tacrolimus. **Figure S2.** Genetic stability analysis of *S. hygroscopicus* SFK-36 by RAPD. M: Marker; G0: RAPD map of parent strain; G10: RAPD map of the strain subcultured for 10 times; RAPD-1, RAPD-2 and RAPD-3: primers were shown at Table S1.

## Abbreviations

ARTP: atmospheric and room temperature plasma
RSM: response surface methodology
RT-PCR: reverse transcription-polymerase chain reaction
qRT-PCR: quantitative real-time polymerase chain reaction
SD: standard deviation
CFU: colony forming unit
PMV: packed mycelium volume
2D: two-dimensional
3D: three-dimensional
RAPD: random amplified polymorphic DNA
PKS: polyketide synthase
