# Supplementary material for: Enhancement of ascomycin production via a combination of atmospheric and room temperature plasma mutagenesis in Streptomyces hygroscopicus and medium optimization
Source: AMB Express. 2019 Feb 18;9:25. doi: 10.1186/s13568-019-0749-x (PMC6379505; doi:10.1186/s13568-019-0749-x)
Supplement: Supplementary file 1 — Additional file 1: Table S1. Sequences of primer pairs used in this study. Figure S1. Structure of ascomycin and tacrolimus. Figure S2. Genetic stability analysis of S. hygroscopicus SFK-36 by RAPD. M: Marker; G0: RAPD map of parent strain; G10: RAPD map of the strain subcultured for 10 times; RAPD-1, RAPD-2 and RAPD-3: primers were shown at Table S1. [file 13568_2019_749_MOESM1_ESM.docx]

**Table S1** Sequences of primers used in this study

| Primer name | Sequence (5’-3’) |
| --- | --- |
| *fkbW-U*-F | AGTGACCGTCCGGGGCATC |
| *fkbW-U*-R | GTTCTTCGGCCTGAGCGGAT |
| *fkbU-R2*-F | GTACATGTTCAGCCGGCCCG |
| *fkbU-R2*-R | AACCAGGACGGTGTCATCGTC |
| *fkbR2*-R1-F | AACCAGGACGGTGTCATCGTC |
| *fkbR2*-R1-R | ACCGAGCTGAGCACGCTGC |
| *fkbF-G*-F | ACGCGAGTCCCTTCTCCACC |
| *fkbF-G*-R | TCCCGATCTCCAGCACCTGA |
| *fkbH-I*-F | TCGTGGTCGTTCTTGCTCGC |
| *fkbH-I*-R | ATGCTGGTCGCGGCGTCAC |
| *fkbK-L*-F | ACCTCGACCGTCGGGATCAG |
| *fkbK-L*-R | CGTTCATCTGCGTCGACCAT |
| *fkbL-C*-F | ACTGCCGCTGTCGTCGTCGA |
| *fkbL-C*-R | GAACTGCTGTCCCCGGACGA |
| *fkbN-Q*-F | ATGGAGCACACCGCGCGAAG |
| *fkbN-Q*-R | ATCGGTGACGACGATCCGAAG |
| *fkbP-A*-F | TTGCACGAAACCGTGCTCGG |
| *fkbP-A*-R | TTCTGGAGTTCCGCGGTCGC |
| *fkbC-B*-F | CCAGGCGATTTCGAGGAGGA |
| *fkbC-B*-R | GGGAACAGGTGCTGCTCGGC |
| *fkbW*-RT-F | ATGGTTCTGCCCCTCGGTGTC |
| *fkbW*-RT-R | AGTGACCGTCCGGGGCATC |
| *fkbU*-RT-F | CGCCGTTCCGGGGAATCC |
| *fkbU*-RT-R | GCCACGAGACGGGTGAGCAC |
| *fkbR1*-RT-F | GTGGTTCACGGCCTTGGCC |
| *fkbR1*-RT-R | ACGCCCACTCGTCGCTCCA |
| *fkbE*-RT-F | GCACGGTGCGTGGCATGT |
| *fkbE*-RT-R | TCCACCAAGGCGTGCAGGT |
| *fkbB*-RT-F | ACGCCGAACTCGCCCACTT |
| *fkbB*-RT-R | AGGAGACGCAGTCCGGTGCC |
| *fkbO*-RT-F | ATGACCGATGCCGGACGC |
| *fkbO*-RT-R | CGTCACCCTCGAGGTCCGGT |
| *fkbS*-RT-F | TGCGTGACCTTCTTCAGGCG |
| *fkbS*-RT-R | ATCGGGGTGCTTCTCATGGG |
| 16S- RT-F | AAGGCGACGACGGGTAGCCG |
| 16S- RT-F | CACTTGCGCTTCTTCCCTGC |
| RAPD-1 | ACTTCGCCAC |
| RAPD-2 | GGTGCGGGAA |
| RAPD-3 | CATCGCCGCA |

**Figure S1**

**R: CH_2_CH_3_ Ascomycin**

**CH_2_CH=CH_2_  Tacrolimus**

**Figure S2**

**
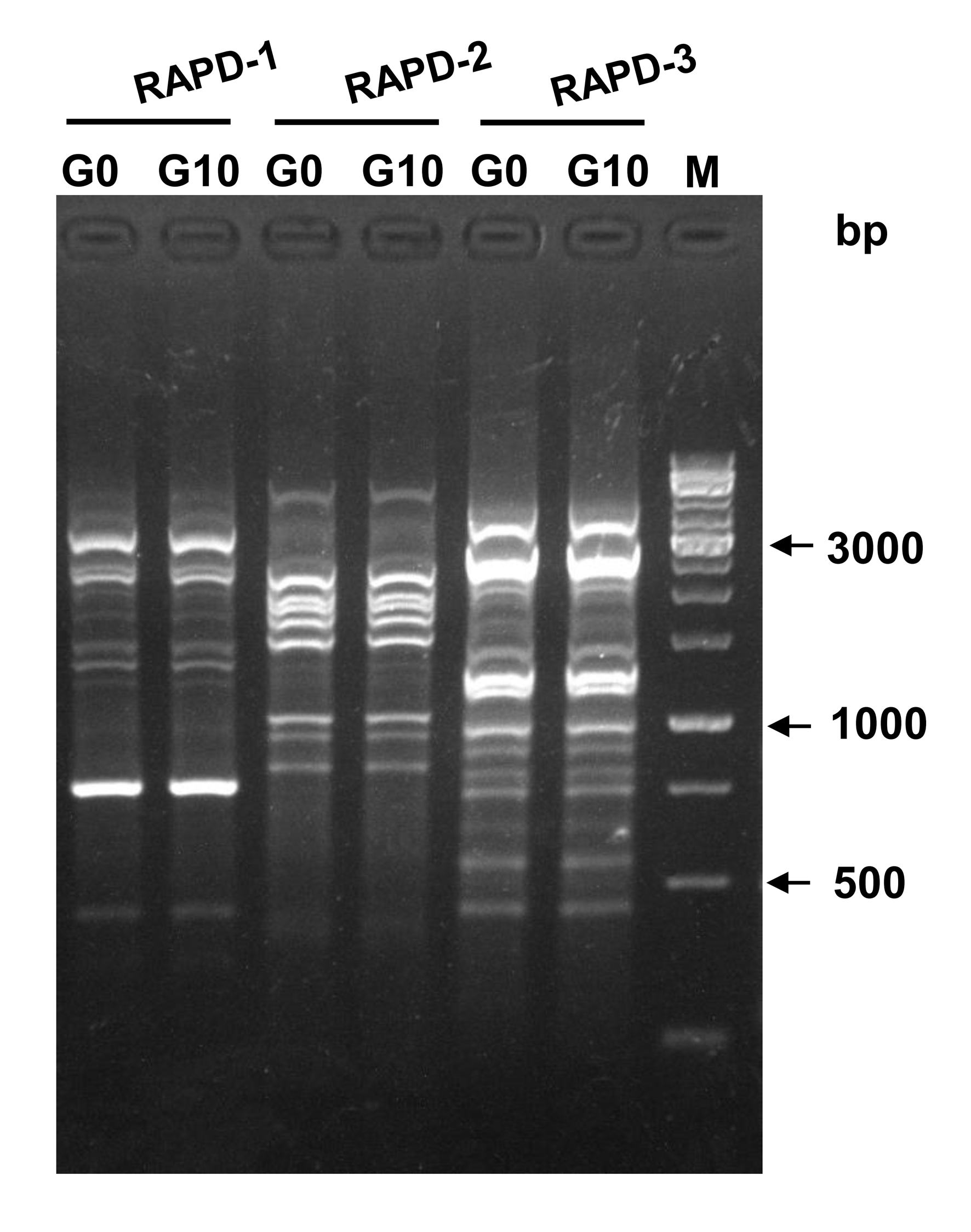
**
